# Supplementary material for: Association of select psychiatric disorders with incident brain aneurysm and subarachnoid hemorrhage among veterans
Source: Front Integr Neurosci. 2023 Aug 2;17:1207610. doi: 10.3389/fnint.2023.1207610 (PMC10433370; doi:10.3389/fnint.2023.1207610)
Supplement: Supplementary file 1 [file Data_Sheet_1.PDF]

**Supplemental Table 1**: ICD and CPT Codes			
	**ICD-9-CM Diagnosis Codes**	**ICD-10-CM Diagnosis Codes**	**CPT Procedure Codes**
**Aneurysm**	437.3	I67.1	61697, 61698, 61700, 61702, 61624
**SAH**	430	I60	
**PTSD**	309.81	F43.10, F43.11, F43.12	
**Depression**	296.2, 296.21, 296.22, 296.23, 296.24, 296.25, 296.26, 296.3, 296.31, 296.32, 296.33, 296.34, 296.35, 311	F32, F32.0, F32.1, F32.2, F32.3, F32.4, F32.5, F32.8, F32.81, F32.89, F32.9, F33, F33.1, F33.2, F33.3, F33.4, F33.41, F33.42, F33.8, F33.9	
**Generalized Anxiety Disorder/GAD**	F41, F41.0, F41.1, F41.3, F41.8, F41.9	300, 300.01, 300.02, 300.09	
**Other mood disorders**	296.4, 296.41, 296.42, 296.43, 296.44, 296.45, 296.46, 296.5, 296.51, 296.52, 296.53, 296.54, 296.55, 296.56, 296.6, 296.61, 296.62, 296.63, 296.64, 296.65, 296.66, 296.7, 296.8, 296.81, 296.82, 296.89, 296.9, 296.99	: F34.0, F34.1, F34.8, F34.81, F34.89, F34.0, F31.0, F31.10, F31.11, F31.12, F31.13, F31.2, F31.30, F31.31, F31.32, F31.4, F31.5, F31.60, F31.61, F31.62, F31.63, F31.64, F31.70, F31.71, F31.72, F31.73, F31.74, F31.75, F31.76, F31.77, F31.78, F31.81, F31.89, F31.9	

Supplemental Table 2. Multivariable Model of Predictors of Aneurysm without Subarachnoid Hemorrhage

Variable	OR (95% CI)	p-value
**Female**	1.74 (1.66-1.82)	<0.0001
**Hypertension**	1.47 (1.43-1.52)	<0.0001
**Diabetes mellitus**	0.96 (0.94-0.99)	0.003
**Tobacco Use Disorder**	1.25 (1.22-1.28)	<0.0001
**Generalized Anxiety Disorder**	1.11 (1.07-1.15)	<0.0001
**Depression**	1.24 (1.20-1.27)	<0.0001
**Other mood disorders**	1.15 (1.09-1.22)	<0.0001
**Post-traumatic stress disorder**	1.24 (1.18-1.31)	<0.0001

**Supplemental Table 3.** Multivariable Model of Predictors of Subarachnoid Hemorrhage		
**Variable**	**OR (95% CI)**	**p-value**
**Female**	1.77 (1.60-1.97)	<0.0001
**Hypertension**	1.65 (1.54-1.77)	<0.0001
**Diabetes mellitus**	0.81 (0.76-0.86)	<0.0001
**Tobacco Use Disorder**	1.49 (1.41-1.59)	<0.0001
**Generalized Anxiety Disorder**	1.03 (0.95-1.13)	0.47
**Depression**	1.34 (1.25-1.43)	<0.0001
**Other mood disorders**	1.14 (1.00-1.29)	0.05
**Post-traumatic stress disorder**	1.10 (0.97-1.24)	0.14
